# In Vitro Release of Interferon-Gamma from Peripheral Blood Lymphocytes in Cutaneous Adverse Drug Reactions

**DOI:** 10.1155/2012/687532

**Published:** 2012-06-06

**Authors:** Ilan Goldberg, Meital Hanson, Gabriel Chodick, Idit Shirazi, Sarah Brenner

**Affiliations:** ^1^Department of Dermatology, Tel Aviv Sourasky Medical Center, Sackler Faculty of Medicine, Tel Aviv University, Tel Aviv 64239, Israel; ^2^School of Public Health, Sackler Faculty of Medicine, Tel Aviv University, Tel Aviv, Israel

## Abstract

*Background*. Cutaneous drug reactions are common but diagnostically challenging due to phenotypic heterogeneity and simultaneous exposure to multiple drugs. These limitations prompted the development of diagnostic tests. *Aims*. To evaluate the performance of an in vitro assay measuring interferon-gamma release from patients' lymphocytes in the presence of causative drugs for the diagnosis of drug reactions. *Methods*. Mononuclear cells derived from patients were incubated with and without suspected drugs, and increment of interferon-gamma levels was measured by ELISA. We performed a telephonic survey to evaluate the effect of stopping the drugs incriminated by the assay on cutaneous manifestations. *Results*. We assessed 272 patients who used 1035 medications. When assessed against the questionnaire data collected at least 6 months after stopping the causative drug, sensitivity was found to be 83.61% and specificity 92.67%. Likelihood ratio for a positive test is 11.40 and for a negative test 0.18. Positive predictive value is 75.37% and negative predictive value is 95.47%. The test was found to perform significantly better in females and in older patients. *Conclusions*. Interferon-gamma release test is a useful adjunct tool in the diagnosis of cutaneous drug reactions.

## 1. Introduction

Cutaneous adverse drug reaction is a common phenomenon consisting of pathological alterations of the structure and/or the function of the skin, adnexa, and mucosa, as a result of exposure to a medication. The plethora of clinical manifestations associated with cutaneous drug reactions significantly complicates clinical diagnosis as well as pharmacological counseling. Indeed, many types of skin reactions to drugs have been reported and many skin reactions may imitate common diseases such as bullous diseases, psoriasis and so forth [1–3]. In addition, very often patients with skin drug reactions have been exposed to multiple drugs and reactions to some drugs are characterized by a prolonged latency period between exposure and the onset of the skin reaction. Finally, numerous cofactors (such as viral infection or autoimmunity) are influencing the propensity to develop allergic rashes to a given medication. To further complicate the matter, associated laboratory findings such as eosinophilia and liver function test abnormalities are nonspecific and therefore unhelpful [4]. 

These facts have prompted the development of numerous specific ancillary assays to assist physicians in the diagnosis and prevention of drug reactions [4].

 In vivo testing consisting in drug rechallenge entails exposure of the patients to potentially harmful consequences of the test and has been largely abandoned [4, 5]. Although in vitro tests are generally technically more cumbersome, they are being increasingly used because of safety considerations and because they can be more easily standardized. Many techniques have been developed which measure various immunological parameters in response to exposure of patients' cells to suspected drugs. These assays include radioallergosorbent tests (RAST) [6], mast cell degranulation test [7], lymphocytic transformation test or proliferation test [8–11], the release of beta glucuronidase from white cells test [12], lymphocyte toxicity assay [13], macrophage migration inhibitory factor (MIF) assay [14, 15], and tests that are based on interferon-gamma (IFN-gamma) release from lymphocytes [5, 16–20].

A large body of in vivo and in vitro evidence indicates that T lymphocytes are involved in the pathogenesis of cutaneous drug hypersensitivity. Drugs can stimulate subpopulations of CD4 and CD8 type T cells [21]. These activated cells secrete different cytokines such as IFN-gamma [16]. Laboratory tests that are based on the release of cytokines from patient's lymphocytes, in response to in vitro incubation with the suspected drugs, may indicate a cellular immune response unique for the drug and indicate the role of the drug in causing the reaction. IFN-gamma is Th1 type cytokine and is an important mediator of the delayed hypersensitivity that acts also as a macrophage activator. In vitro demonstration of IFN-gamma release from lymphocytes after an in vitro incubation with a suspected drug has been shown to be of diagnostic value in skin reactions based on the demonstration of cutaneous rash resolution upon cessation of the drug incriminated by the in vitro assay [5]. Unfortunately, much of the data supporting the use of in vitro assay to diagnose cutaneous drug reactions are based on short-term followup of the patients. The aim of the present study was to determine the performance of the IFN-gamma release assay when assessed against the data of long-term patients followup.

## 2. Methods

### 2.1. Study Population

The study was approved by the Medical Center Ethics Committee. We assessed all patients with cutaneous adverse drug reactions who underwent a routine IFN-gamma release testing at our department between the years 2003–2007.

Clinical data were collected from the patient's medical files and from a telephonic interview with the patient. The details collected included demographic data (age, sex), type of skin reaction, suspected drug, whether the treatment with the drug was stopped following the test, whether there was an improvement in the state of the rash, whether the rash relapsed, was there an event that preceded the relapse of the rash, whether a different diagnosis was found for the rash and was it treated.

Clinical improvement attributable to drug cessation was defined when the patient reported one of the following:

- Improvement of the rash without relapse, upon cessation of the drug.
- Improvement of the rash upon cessation of the drug and relapse of the rash as the patient renewed the drug.
- Improvement of the rash upon cessation of the drug and relapse of the rash as the patient started taking a drug structurally related to the drug that was stopped.

Absence of clinical improvement attributable to drug cessation was defined when the patient reported one of the following:

- There was no clinical improvement of the rash after the patient stopped using the drug.
- There was improvement in the rash although the patient continued using the suspected drug.
- There was improvement in the rash upon cessation of the drug and relapse of the rash although the patient did not renew the drug.
- Clinical improvement followed another diagnosis and a specific treatment for that diagnosis.

**Figure 1:**
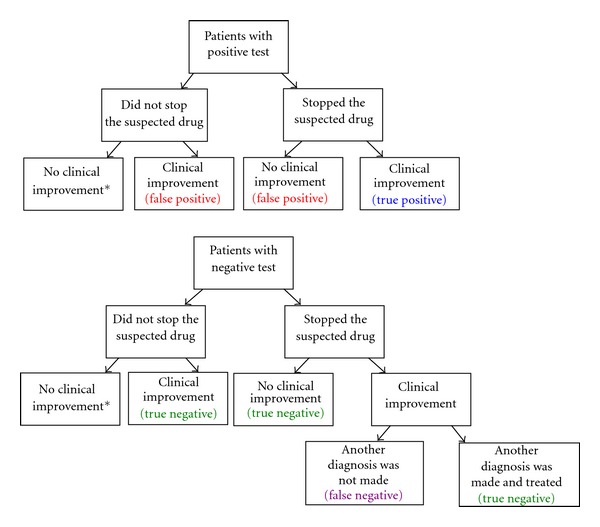
Division of study population. *It cannot be determined whether continuing the suspected drug is indeed responsible for the continuing clinical symptoms. **It cannot be determined whether the drug that was found negative in the test is indeed not responsible for the clinical symptoms.

Based on these data, we divided our population into 4 groups (Figure 1):

Group A: true positive,

Group B: false positive,

Group C: true negative,

Group D: false negative.

We also divided our population into 5 groups according to the type of the rash:

Group 1: patients with urticaria,

Group 2: patients with morbilliform rash,

Group 3: patients with psoriasis/psoriasiform eruption,

Group 4: patients with vasculitis,

Group 5: patients with other skin reactions.

### 2.2. IFN-Gamma Release Test

Lymphocytes were separated from heparinized venous blood by Ficoll-Hypaque gradient centrifugation and cultured for 24 hours in 24 well plates containing Dulbecco's Modified Eagle's medium (Biological industries, Beit Haemek, Israel), phytohemagglutinin (Biological industries, Beit Haemek, Israel), in the presence and the absence of the drug. Following incubation for 24 hours in 5% CO_2_ at 37°C and centrifugation at 2500 rpm for 25 min at 5°C, the supernatants were collected for the detection of IFN-gamma using a standard ELISA assay (Biosource, Enco Diagnostics, Petach Tikvah, Israel). IFN-gamma release was expressed as follows: 100 × (IFN-gamma with the drug − IFN-gamma with medium alone)/IFN-gamma with medium alone.

A positive test was defined as corresponding to a value of more than 130%. A borderline test was defined as a value between 120%–129% [4].

### 2.3. Statistical Methods

Calculation of sensitivity, specificity, likelihood ratio, and 95% confidence intervals was made using a standard statistical program (WINPEPI for Windows) [22].

Evaluation of odds ratio (OR) and confidence intervals for clinical improvement according to drugs usage and the test's results was made using logistic regression model with adjustments to age and sex (SPSS for Windows).

## 3. Results

284 patients were assessed in this study. In 12 (4.25%) patients, we could not retrieve all information data due to the death of the patient or lack of patient's ability to answer our questions. Thus, all subsequent calculations were obtained using the set of 272 remaining patients. Altogether, these patients were found to receive a total of 1035 medications prior to the onset of the cutaneous rash. These medications included antibiotics (7%), analgesics (20%), angiotensin-converting enzyme inhibitors (4%), statins (7%), and others. Of all 1035 drugs assessed, a definite (positive or negative) result for the IFN-gamma release test was obtained in 953 (92%). In 82 (7.9%) of the medications, the test result was borderline (a borderline test was defined as corresponding to an IFN-gamma release value between 120%–129%). Data were processed after excluding 105 medications for which the test results were without attribution to a study group (medications for which it was impossible to determine whether the continuation of the suspected drug was responsible for the continuation of the clinical symptoms or that it was impossible to determine whether the drug testing negative by the IFN-gamma release assay was indeed not responsible for the cutaneous symptoms). Of the 930 medications left, 865 (93%) were associated with a definite IFN-gamma release assay result (785 true positive/negative results and 80 false positive/negative results) for which it was possible to compare clinical outcome to test result (Table 1).

Sixty-five (7%) of the drugs tested borderline in the assay and were classified as either positive borderline (*n* = 20; a borderline result in the assay with clinical evidence for a causative role of the drug in the cutaneous reaction) or negative borderline (*n* = 45; a borderline result in the assay for which no correlation between the test result and the clinical course was found).

The sensitivity and specificity were determined based on the definite results; borderline and nonclassifiable assays were excluded from the analysis.

Sensitivity and specificity of the assay were 83.61% (C.I. 95%: 77.56% −88.27%) and 92.67% (C.I. 95%: 90.46%–94.39%), respectively. Likelihood ratio for a positive or a negative test was 11.40, (C.I. 95%: 8.67–15.01) and 0.18, (C.I. 95%: 0.13–0.25), respectively. The positive predictive value of the test is 75.37% (C.I. 95%: 69.95–80.09%) and its negative predictive value is 95.47% (C.I. 95%: 93.83–96.69%).

Influence of age and sex on the performance of the IFN-gamma release test was assessed using a multivariable logistic regression model, in which the dependent variable is a true or false result (Table 2).

As shown in Table 2, age was significantly associated with a true positive/negative result in the IFN-gamma release test. Every additional year of age was associated with a 1.6% increase in the probability of a true result. Similarly, female sex was associated with a significantly higher rate of true positive/negative result (*P* = 0.027). Odds ratio of a true result in men was 41.5% lower as compared with women.

Among patients who display vasculitis, the probability of a true result was slightly (but not significantly) (*P* = 0.08) higher than for patients affected by an urticarial rash. There was no statistically significant difference between the rash groups.

The specific effect of age, sex, and type of skin reaction on test performance is presented in Tables 3, 4, and 5, respectively. The test's sensitivity, specificity, and positive and negative likelihood ratio derived from these data are presented in Table 6.

## 4. Discussion

As discussed above, cutaneous drug reactions are often diagnostically very challenging. To our knowledge, the present data provide, for the first time, evidence based on long-term follow-up data that an in vitro assay may represent a useful adjunct to the clinical diagnosis of this common dermatological occurrence. This is of particular importance when the morphological features of the rash overlap with those of a common drug-unrelated skin eruption (e.g., psoriasis). In addition, when a patient is taking a number of drugs simultaneously, in vitro ancillary assays can help pointing out the culprit drug and avoid unnecessary withdrawal of essential medications.

The IFN-gamma release assay is based on the involvement of T lymphocytes in the pathogenesis of cutaneous adverse drug reactions. Drugs stimulate subpopulation of CD4+ and CD8+ type T cells, with Th1 or Th2 cytokines pattern, according to the drug and the drug reaction type [21]. Reactions associated with delayed hypersensitivity are characterized by preferential activation of Th1 cells. In contrast, drug eruptions resulting from immediate hypersensitivity are characterized by a Th2 reaction pattern. Interestingly, although IFN-gamma is typically categorized as a Th1 cytokine, high levels of this molecule have been detected in patients with immediate hypersensitivity reactions [23].

In this study, sensitivity, specificity, positive and negative predictive values of an IFN-gamma release assay were found to be high for the diagnosis of cutaneous skin reactions.

Previous studies have similarly examined the efficacy of this test and their results are in line with the present data. However, this study examined the reliability of the test results over a long period of time as patients were interviewed at least half a year after the test was performed, in order to find out if there was a relapse of the rash after the cessation of the drug, or whether the patient continued to take the drugs without a rash, and thus knowing retrospectively whether the test result was true or false.

65 (7%) of the drugs had a borderline result in the test (45 of them were retrospectively found to be clinically negative and 20 clinically positive). Although most of the borderline results were found to be clinically negative, it is still important to define for this test a range of borderline results (between 120%–130% increase in IFN-gamma release), in order not to miss cases in which the drugs are indeed responsible for the skin reaction. In patients for whom all drugs are tested negative and/or borderline, it is recommended to avoid using the borderline drugs.

Livni et al. examined the efficacy of the test in patients with urticaria and angioedema. They found that the test's sensitivity was 50% and its specificity 92%. They also compared the IFN-gamma release assay with the MIF assay and found 80.9% agreement between the two tests [19]. In a study that examined the test's efficacy in cases of allergy to potassium dichromate in 20 allergic patients and 30 control individuals, the assay's sensitivity was 73.7% and its specificity 71.4% [24]. In another study of 36 patients with cutaneous adverse drug reactions, the test was found to be characterized by a sensitivity of 77.8% [16].

False positive results can result from many causes including performance of the assay during the acute phase of the rash, cross reactivity or multiple drug allergy syndrome or sensitivity to a preservative that is found in a number of drugs [16]. Non-IFN-related mechanisms may underlie cutaneous drug reactions for which false negative results are obtained. As mentioned above, there are skin reactions caused by nonimmunological mechanisms or characterized by cytokine release patterns that do not involve interferon secretion [25]. Another reason for false negative results is the fact that in some cases drug metabolites or haptenized drugs rather than the native drug are responsible for the pathological reaction [26–28]. Photosensitive reaction is another example of a drug allergy that may not be reflected in an in vitro assay [29]. An additional reason for false negative results is a test performed during a corticosteroid or other immunosuppressive treatment.

It should be noted that, in this study, in most of the patients for whom a drug that caused the rash was identified, the other drugs that the patient used tested negative (even if the patient used more than 10 drugs), underscoring the usefulness of this assay in enabling continued administration of important drugs.

In our study we found that the test performed significantly better in women than in men (*P* = 0.027). This is in agreement with another study, recently published by Saito et al. which compared between the efficacy of the leukocyte migration test (LMT) and of the lymphocyte stimulation test (LST) to assess drug sensitivity. In that study it was found that women had significantly more positive results than men for the LMT assay, which may be related to the fact women produce higher levels of cytokines and chemokines from lymphocytes than men [30].

Our study also revealed that older people were more likely to have true positive/negative results than younger people. Saito et al. did not report significant difference in the results by age, although they mentioned such differences in their previous study [30]. This finding is somewhat difficult to interpret and it may be related to the type of drugs that the older population uses in comparison to younger patients, and to the types of rash and their influence on the cytokine pattern that is produced by the lymphocytes.

When we assessed the patients by the type of rash, we found that patients with vasculitis showed a trend (*P* = 0.08) for higher sensitivity and specificity values (100% and 96.97% resp.), in comparison to patients in other groups (Table 6). In another study that examined the performance of the IFN-gamma release test, a high increase in IFN-gamma release was found among patients with vasculitis, and the test was found positive in 100% of patients with vasculitis [16].

In conclusion, in this study we examined the performance of the IFN-gamma release test as assessed against data collected from a large number of patients over a prolonged period of time. The results of the study indicate that this assay can serve as a useful adjunct in the diagnosis of cutaneous drug reactions.

## Figures and Tables

**Table 1:** Correlation between clinical course and IFN-gamma release results for all medications.

Assay results	False result according to questionnaire	True result according to questionnaire	Total medications
Negative	30	632	662
Positive	50	153	203

Total	80	785	865

**Table 2:** Influence of patient age and sex on the performance of the IFN-gamma release test.

	Number of medications taken by the patients	*P*	Odds ratio for true result (95% confidence intervals)
Sex (men versus women),	M: 316 medications, W: 549 medications	0.027	0.585 (0.364–0.940)
Age (per year)		0.006	1.016 (1.004–1.028)

**Table 3:** Correlation between patient's sex and IFN-gamma release results for all medications.

Sex	Test result	False result according to questionnaire	True result according to questionnaire	Total drugs
Women	Negative	16	395	411
	Positive	28	110	138
	Total	44	505	549

Men	Negative	14	237	251
	Positive	22	43	65
	Total	36	280	316

**Table 4:** Correlation between patient's age and IFN-gamma release results for all medications.

Age group (years)	Test result	False result according to questionnaire	True result according to questionnaire	Total drugs
≤44	Negative	9	155	164
	Positive	20	52	72
	Total	29	207	236

45–64	Negative	14	181	195
	Positive	17	44	61
	Total	31	225	256

≥65	Negative	7	296	303
	Positive	13	56	69
	Total	20	352	372

**Table 5:** Correlation between vasculitis patients (group 4) in comparison to other patients and IFN-gamma release results for all medications.

Rash	Test result	False result according to questionnaire	True result according to questionnaire	Total drugs
Other groups	Negative	30	600	630
	Positive	49	141	190
	Total	79	741	820

Group 4	Negative	0	32	32
	Positive	1	12	13
	Total	1	44	45

**Table 6:** Sensitivity and specificity of the IFN-gamma release test, and positive and negative likelihood ratio according to patient's age groups, sex, and type of rash.

Age group/sex	Sensitivity (%) 95% CI (range)	Specificity (%) 95% CI (range)	Likelihood ratio for a positive test 95% CI (range)	Likelihood ratio for a negative test 95% CI (range)
≤44 years	85.25 (74.28–92.04)	88.57 (83.01–92.48)	7.46 (4.87–11.41)	0.17 (0.09–0.13)
45–64 years	75.86 (63.47–85.04)	91.71 (87.12–94.76)	9.15 (5.67–14.75)	0.26 (0.17–0.42)
≥65 years	88.89 (78.8–94.51)	95.79 (92.94–97.53)	21.3 (12.32–36.23)	0.12 (0.06–0.23)
Women	87.30 (80.37–92.03)	93.38 (90.6–95.38)	13.19 (9.16–18.98)	0.14 (0.09–0.22)
Men	75.44 (62.9–84.77)	91.51 (87.47–94.32)	8.88 (5.8–13.6)	0.27 (0.17–0.42)
Vasculitis	100 (75.75–100)	96.97 (84.68–99.46)	33.0 (4.79–37.7)	0.00
All other skin reactions	82.46 (76.06–87.43)	92.45 (90.16–94.24)	10.9 (8.27–14.42)	0.19 (0.14–0.26)
